# Advice for Elderly Drivers in a German Memory Clinic: A Case Report on Medical, Ethical and Legal Consequences

**DOI:** 10.3390/geriatrics1010009

**Published:** 2016-03-19

**Authors:** Stefan Spannhorst, Max Toepper, Philipp Schulz, Gudrun Wenzel, Martin Driessen, Stefan Kreisel

**Affiliations:** Division of Geriatric Psychiatry, Department of Psychiatry and Psychotherapy Bethel, Evangelisches Krankenhaus Bielefeld, Bethesdaweg 12, D-33617 Bielefeld, Germany; Max.Toepper@evkb.de (M.T.); Philipp.Schulz@evkb.de (P.S.); Gudrun.Wenzel@evkb.de (G.W.); Martin.Driessen@evkb.de (M.D.); Stefan.Kreisel@evkb.de (S.K.)

**Keywords:** dementia, driving advice, memory clinic, cognitive decline

## Abstract

We report on a 75-year-old female who consulted our Memory Clinic because of subjective memory complaints that she first recognized three months previously. Next to the standard detailed patient history, neuropsychological assessment, psychopathological status, the patient’s driving history played an important role in the diagnostic process. In this case report, we illustrate the diagnostic process starting with the first consultation, including a short neuropsychological examination and communicating its results, reporting on further work-up (detailed neuropsychological assessment, MRI scan and cerebrospinal fluid (CSF) analysis) up to the final consultation, including advice for the patient. We will focus on several medical, ethical and legal difficulties that may occur when consulting elderly drivers with initial cognitive decline.

## 1. Introduction

The Memory Clinic of the Department of Psychiatry and Psychotherapy Bethel at the Evangelisches Krankenhaus Bielefeld (EvKB) has a focus on traffic safety issues for elderly drivers.

In order to understand the medical, ethical and legal dilemmas in this case report, it is crucial to give some background information first. After presenting general aspects of driving in old age, we illustrate different national jurisdictions and outline the specific workflow in our Memory Clinic.

### 1.1. Driving Ability, Ageing and Cognitive Deficits

Statistically, older individuals (aged 65 and above) are less involved in road accidents than younger drivers [1,2,3]. The number of accidents per kilometer driven, however, is high among seniors [1,4], probably due to the so-called “low mileage bias”: older drivers tend to reduce their driving range and therefore lack routine in driving. Other factors that are associated with accident rates are certain driving behaviors. In fact, long distance drivers usually drive on motorways, which are known to be the safest roads, and, therefore, gain a lot of safe kilometers. In contrast, drivers who mainly use urban roads with more complicated traffic situations and drive less are at higher risk of accidents independent of their age [4,5]. Accordingly, both quantitative and qualitative differences in driving behavior are associated with the accident risk among older drivers. However, the question remains whether reduced driving range is caused by personal choice or social circumstances alone, or whether it may at least partly reflect early changes in driving behavior in terms of self-regulation. In fact, the reduction of driving range or velocity may be signs of a compensation strategy for initial cognitive decline.

Notably, even dementia does not automatically reduce driving fitness, at least not in early disease stages [6]. For medium stage dementia, however, there is no defined cut-off either that could provide the basis for a ban on driving [7], which is surprising since there can be no doubt that dementia eventually leads to impaired driving fitness. Consequently, specific symptoms of cognitive decline and their effects on driving safety are brought into focus. German guidelines recently published by The German Society of Psychiatry, Psychotherapy, Psychosomatics and Neurology (DGPPN) [7] underline the importance of certain domains of cognition for driving fitness. Besides orientation, reaction time and alertness, the ability to cope with complex situations is a necessary precondition for safe driving. Reduction of these abilities and additional difficulties with spatial vision may result in reduced driving skills. In addition, reduced inhibition of automatic reactions can lead to reduced driving safety, as can be seen in patients with Lewy body dementia (DLB) and Parkinson’s dementia (PDD) [8,9]. Patients with frontotemporal dementia often show an inhibitory dysfunction as well, leading to risky driving habits already in early stages of the disease. [6] Grace and colleagues found distinct neuropsychological deficits investigating patients with Parkinson’s disease (PD) and Alzheimer's disease (AD) by relating cognitive and on-road performances [9]. Results showed that the AD group frequently made operational errors such as hesitant driving and diminished awareness of the traffic environment. Moreover, they made more tactical errors at changing lanes, for example, and strategic judgment errors such as driving into a one way street. PD drivers made more tactical errors regarding head turning such as not checking blind spots or pulling out into traffic. In both patient groups, visuospatial and executive deficits were particularly associated with reduced driving performance [9]. Yamin and colleagues found significantly worse driving simulator performances of drivers with mild DLB compared to healthy controls [8], which also appears to be highly relevant since most drivers diagnosed with DLB continue to drive for four years following diagnosis [10]. Ernst and colleagues illustrated the relevance of a closer look at the individual stage of dementia, not on the diagnosis alone [6]. They showed that 40% of individuals with moderate AD have unchanged driving behavior compared to those without dementia. Notably, the rest did show significant driving difficulties that were mainly related to orientation deficits. In contrast hereto, patients with fronto-temporal dementia showed fewer cognitive deficits, yet had disturbed driving skills mainly due to more impulsive behavior. Consequently, cognitive deficits alone do not seem to cover the whole range of difficulties. In fact, some studies suggest that driving problems among elderly patients might be better predicted by avoidance strategies (e.g., not driving at nighttime or in rain) [11].

However, other studies suggest a remarkable predictability of driving performance depending on specific cognitive measures. Classen and colleagues [12], for example, reported that test failure regarding on-road-testing could be predicted by performance in the trail making test B (TMT-B), older age as well as some neurological disorders. Moreover, the Useful Field of View (UFOV) test, a complex reaction time task, a paper folding task, Dot counting and Wechsler Memory Scale Visual Reproduction as well as a computerized visual attention task appeared to be good predictors of on-road driving [13]. Lundberg and colleagues investigated older drivers that had lost their licenses as a consequence of crashes and compared their cognitive performances with the performances of healthy controls [14]. The results revealed deficits in visuospatial functions and in the integration of perception and motor functions in people who had experienced crashes. Finally, old age and changes in motor functions seem to be independent predictors of driving faults [15].

In contrast, Martin and colleagues suggested that neuropsychological results alone never provide sufficient reason for driving cessation [16]. In this context, Bédard and colleagues showed that several predictors (*i.e.,* the Mini Mental Status Examination (MMSE), Trail making test A, UFOV and composite measure of past driving incidents) had just limited predictive value for driving performance, because a statistical correlation between lower test results and reduced driving performance does not necessarily imply a causal relation [17]. Consequently, driving performance must be seen as multifactorial and the analysis of only a few test variables may lead to false conclusions.

Taken together, there are many factors that may impede driving fitness as well as different ways to deal with this problem. Most authors stress the importance of in-depth individualized assessment and regular monitoring of demented patients by professionals once that cognitive deficits occur [8,18]. In fact, it seems that an individualized, sensitive approach to objectify driving-related risk factors might be the most adequate way of dealing with this topic [19,20]. Such approaches should particularly focus on the patient’s individual situation, symptoms and cognitive functions providing the basis for optimal advice.

### 1.2. Different National Approaches to the Topic of Driving Fitness

Jurisdictions regarding driving fitness in older individuals vary across different countries. Some of them have implemented a mandatory driving test for older individuals. European countries practice a wide range of car driving license renewal procedures. These range from issuing lifelong licenses without subsequent medical checks (like in Germany) to issuing license to age 70 and three- or five-year periods, thereafter—based on self-declarations of medical fitness. On the other end of the scale, some countries require medical examinations for renewal or renewal every five years from the age of 45. In a case study, Mitchell from the UK Transport Research Laboratory investigated all mentioned strategies represented by studying assessment policies in France, the Netherlands, United Kingdom, Denmark, Finland, Norway and Sweden in 2008 [20]. The results show that there is no evidence that any license renewal procedure or requirement for medical examination has an effect on the overall safety of drivers aged 65 and older. Keall and Frith focused on the regulations in New Zealand [21]. From May 1999, a new system for licensing older drivers had been introduced. It includes practical on-road-driving test with expanded scope, to be completed every two years from the time the driver turns 80. Results revealed that each driving test failure was associated with an elevated risk of crash by 33 percent controlling for age, gender, minor traffic violations and whether the older driver lived with another licensed driver or not. In Australia, most licensing jurisdictions currently employ an age-based assessment program. Fildes and colleagues found no evidence that this program has safety benefits [22]. Studies focusing on the USA regulations even found that states with age-based screening had more accidents among older drivers [23]. Rock found that, in the state of Illinois, change of license renewal rules had no influence on road safety [24]. The change implied more lenient rules for individuals aged 69 to 74 by removing on-road-testing and stricter rules for individuals aged 80 and above. These drivers had to undergo more frequent checks than before.

The usefulness of age-related relicensing procedures is an ongoing matter of discussion. In summary, there is variability in the studied populations, especially in the age of participants, showing a lack of convincing evidence. In the authors’ opinion, this is one of the reasons why recent strategic discussions tended to focus on the need for individualized screening and a sensitive therapeutic approach with the driver. However, it needs to be highlighted that the patient-physician relationship can be influenced by local contextual factors in terms of jurisdiction or legal framework for physicians to report on unfit drivers to licensing authorities. In the former scenario, patients may then have a reduced tendency to hide their symptoms to their doctors, which enables a more coaching style of approach by the doctor rather than a judgemental one. Nevertheless, establishing a trustworthy relationship that allows the discussion on the topic of the risks associated with impaired driving remains challenging.

### 1.3. Jurisdiction and Guidelines in Germany

In Germany, the driving license never expires automatically. There are no age-related tests or formal requirements for older drivers. Official German regulations (“Fahreignungsverordnung”) only claim that certain diseases *might* limit driving skills [25]. As a consequence, General Practitioners (GPs) and specialists must rely on vague hints regarding possible risk factors. By law, indeed, the driver is forced to decide critically on himself whether driving is safe or not. The only exception is a medical or medico-psychological official report that is indicated in cases of alcohol or drug consumption with obvious influence on driving skills (as stated by police). Official reports may also become mandatory in cases of other driving offences as hit-and-run offences. In these cases, traffic administration can force the driver to undergo an official report in licensed traffic institutions including examinations performed by certified traffic psychologists and physicians. The specific procedures in such reports (e.g., test batteries) are regulated by guidelines [25]. In clinical everyday practice, however, GPs and specialists have no official guidelines, particularly not for demented patients. In addition, there is a lack of guidelines helping to advice elderly adults with cognitive deficits.

German law implies medical confidentiality that may only be violated in cases of immediate danger for the patient or third parties. There is no right to inform police or traffic administration about an increasing cognitive deficit in spite of its underlying possible danger. This situation leads to a focus on a well-balanced objective advising procedure among professionals who treat older adults. It needs to respect both freedom and safety of the individual patient. A special driving assessment as provided by occupational therapists in various countries (e.g., USA, Australia, Canada and Singapore) is not available in Germany, although there is evidence for enhanced coping and quality of life with occupational therapists assisting in driver retirement [2]. In Germany, for safety and legal reasons, accompanied driving is only possible in the presence of two professionals (plus patient). This means that a traffic psychologist and a driving instructor must accompany the patient and that on-road-testing is only allowed when using a car with specific technical features (*i.e.,* additional pedals, driving school car). Moreover, a neuropsychological examination is mandatory before driving whenever any deficits become obvious. If the patient fails this examination, it is illegal to perform on-road-testing. In Germany, even official road safety institutions primarily rely on the percentile rank 16 criterion to test driving fitness, and not primarily on practical testing. The percentile rank 16 criterion assumes that fitness to drive is only given if individuals reach a percentile rank of at least 16 in all subtests of the respective test battery. One example of a licensed test battery is the “Wiener Testsystem” [26]. Taken together, these circumstances make it difficult to provide on-road-testing for a bigger population in Germany. Moreover, on-road testing requires immense personnel and financial resources that go far beyond clinical routines. These conditions highlight the need for the development of alternative instruments that guarantee an objective but economic assessment of driving fitness. Based on the recommendations of a consensus paper published by Swiss memory clinics [19], we therefore developed the Safety Advice For Elderly drivers (SAFE) questionnaire covering different risk factors for driving safety [27]. The first part of this questionnaire includes questions about the patient’s driving history and basic activities of daily living. Moreover, it covers visual and motor risk factors as well as specific disorders, symptoms and medication that may affect driving fitness. The second part of the SAFE comprises specific neuropsychological test results (*i.e.*, TMT-B, MMSE) as well as dementia etiology and severity. The risk factors are summed up leading to an overall risk estimation ranging from low risk to high risk on a Likert scale.

### 1.4. Workflow of Investigations in Our Memory Clinic

Importantly, the indication for individuals to start investigation in our Memory Clinic are suspected memory deficits. Questions about driving safety are secondary although it is the responsibility of physicians to adequately advise their patients regarding driving safety [28].

Generally, our workflow comprises three up to four appointments with the patient. The first appointment consists of a consultation with an experienced psychiatrist. He examines the medical history of the client including examination of orientation and memory (*i.e.*, MMSE) as well as the patient’s psychopathological status. He adds questions about driving performance including limitations of driving as indicated by the SAFE. In most cases, patients are accompanied by relatives. Thus, the psychiatrist is able to get objective information about the patient’s medical history. At the end of this first consultation, the psychiatrist decides about the indication for further neuropsychological examination. Sometimes, he additionally refers to the patient to a cerebral Magnet Resonance Imaging (MRI )scan and/or lumbar puncture to gain CSF. In some cases, a recent MRI finding is available.

The second appointment comprises a detailed neuropsychological examination by a certified neuropsychologist (see below). After this examination, results are discussed in a weekly multiprofessional consensus conference including a psychiatrist, a neuropsychiatrist (one of them has led the first appointment), the clinical neuropsychologist and two social workers. In this meeting, SAFE is complemented by the neuropsychological findings and possible diagnoses based upon psychopathological findings and cerebral imaging if available. In addition to a consensual judgement, possible consequences for the patient are discussed, including driving fitness. In some cases, the diagnosis remains unclear and a repetition of neuropsychological examination is recommended. Two social workers focus on alternative transportation facilities analyzing the family situation and location of the patients’ social environment. The diagnosis and its implications, possible further diagnostic steps (like cerebrospinal fluid examination) and advice for driving (cessation) are documented to be available for the treating psychiatrist during the third appointment.

The third appointment with the patient comprises information about the results of the neuropsychological examination. It also includes detailed advice concerning future driving. In cases of dementia and/or clear evidence for impaired driving fitness, one of the social workers takes part in the consultation automatically. She offers further psychoeducation for patient and family and stresses possible alternatives of mobility like public transport. She provides information about bus routes and gives practical advice. She also testifies the ban on driving by the psychiatrist. Depending on the complexity of the case and the need for in-depth consulting, we sometimes offer a fourth appointment with the social worker and/or psychiatrist.

### 1.5. Conclusions

Demographic change—not only in Western societies—will lead to an increasing number and percentage of elderly drivers. A decreasing number of driving licenses among younger individuals in Germany contribute to this effect [29]. Younger individuals tend to use public transport and increasingly abstain from getting a driving license. While most older drivers will remain without severe cognitive deficits, the number of individuals with neurodegenerative disorders will increase. As such the topic at hand—providing such patients with reliable consultation—will become more important in the years to come.

It has been shown that older drivers are able to compensate reduced motor skills, reaction times and cognition by their driving experience—at least to some extent [19]. Unfortunately, there is a dearth of studies quantifying the extent to which such compensation of driving limitations is in fact possible. On the other hand, attenuation in driving will lead to reduced training and as a consequence may cause inflexibility, especially in complex traffic situations [14,15].

With respect to the main group of patients in our Memory Clinic—drivers and non-drivers with initial cognitive decline or dementia—studies show a diversity of possible consequences for driving abilities, mainly depending on the kind and severity of cognitive deficits. The fact that patients seeking help in a memory clinic do so primarily because of memory complaints and not because of associated driving difficulties is a challenge. Accordingly, they do not expect to face advice that includes giving up driving. The situation may be further complicated by the fact that, under German law, medical confidentiality is paramount. Only under the most extreme circumstances will it be possible to inform third parties such as relatives or the police, given inherent risk to oneself or others. Fortunately, many patients are accompanied by their relatives or allow the physicians to inform the family about the results of work-up.

Thus, there is potential for ethical dilemma—weighing the medical responsibility that we have for the patient, the obligation to inform him or her if deficits are present and to safeguard the public from danger arising from these deficits. Next to this ethical tightrope, the question of safe participation in road traffic is often a taboo topic in consultation between (elderly) patients and their physicians, given that it can be a highly emotional topic. General practitioners (GPs) may have no interest in querying, not wanting to endanger the therapeutic relationship.

In Germany, active participation in road traffic is commonly regarded as a “right”—the driving license having been “legally” acquired for life, irrespective of possible later disorders limiting driving abilities. The emotional bond some have with their own cars can be large, to say the least, it often being seen as a status symbol, guaranteeing mobility even in later life.

There may also be therapeutic dilemma, perhaps best documented in an example of patients without dementia. Elderly depressed patients may be instructed to refrain from driving under psychopharmacological medication. At the same time, a depressed patient could be advised to foster his or her social contacts, this then becoming impossible if other means of transportation are limited.

In summary, consultation regarding driving safety is by no means an easy endeavor, confounded by following constraints

- Medical: Especially given “borderline” impairment, there remains uncertainty as to the diagnosis and, consequently, the prognosis.
- Ethical: The impact of advice on patients and their loved ones must be critically questioned
- Legal: The impact of limitations in driving safety (*i.e.*, an increased risk) on others has to be discussed in light of confidentiality issues.

The following case report of a 75-year-old female consulting our Memory Clinic allows these points to be discussed practically. The patient provided a written declaration of consent. All examinations had clinical reasons, none of the procedures exceeded standard diagnostics. All procedures were performed in accordance with the Declaration of Helsinki.

## 2. Case

Mrs. L., 75-year-old female patient, consulted our Memory Clinic in 2015 and underwent usual diagnostics in order to objectify or rule out cognitive deficits pointing toward possible dementia. Mrs. L. underwent neurological and psychiatric as same as neuropsychological examination, brain imaging and lumbar puncture, spread out across several appointments as is common in this outpatient setting. Mrs. L. was informed about the results, including advice regarding possible driving limitations.

Note that results of the work-up are routinely discussed in a multiprofessional team during a consensus meeting including physicians, social workers and a clinical neuropsychologist.

### 2.1. Patient History

#### 2.1.1. History

Mrs. L. reported a history of forgetfulness, starting approximately three months previous to the appointment.

She stated that she needed “to write down everything” in order not to forget things. Previously, she had only used notes when shopping. Being among her family and friends, she often forgot the content of the conversation. She complained about forgetting what she wanted to fetch when going to the cellar. She said that she was able to keep house with a little help from her partner, whom she first met only four months ago. Her husband had died two years previously. She did not report difficulties with spatial orientation, sleep, appetite or motivation.

She had noticed an increasing inner restlessness combined with a fear of having dementia (*i.e.*, subjective cognitive impairment). This was the reason for a prescription of 20 mg of Citalopram as antidepressant medication by her GP—the medication was continued up to consultation with us. Further questioning in our Memory Clinic did not reveal signs of depression, nor for, e.g., delirium or any other acute psychiatric illness.

Mrs. L. added that she still liked to go out with friends for ninepins, although less frequently in the last months. She underlined that she liked driving very much and never had difficulties as a driver.

#### 2.1.2. Personal History

Mrs. L. completed secondary education and worked as a seamstress. She has one daughter and was widowed two years ago. Meanwhile, she lives in a house together with her new partner.

There is an unclear history of drinking alcohol: Mrs. L. reported stopping “relevant” drinking four months ago, recently consuming one glass of beer per week, without providing further details.

#### 2.1.3. History Reported by Her Partner

He noticed that Mrs. L. repeatedly asked for the newspaper on Sundays, obviously unable to remember that there never was a newspaper on Sundays. She had expressed her feeling of loneliness since the death of her husband and that she suffered from this. The question of having dementia had become more prominent in the preceding weeks. He never noticed increased alcohol consumption.

#### 2.1.4. History Reported by Her Daughter

The daughter of Mrs. L. suggests regular alcohol consumption (unclear amounts) and expresses her fears of seeing her mother driving, not knowing whether she had drunk recently. She confirms the history given by her mother and the partner (see below for the patient’s history on substance abuse.)

#### 2.1.5. Medical History

No “known” psychiatric illness. Thyroid disease is present, and the patient is on medication. She is also being medically treated for hypertension. No other illnesses were reported.

#### 2.1.6. Driving History

She obtained her driving license 40 years ago. No relevant accidents are reported. Her fellow passengers have felt safe when accompanying her. Starting approximately two years ago, she avoids driving at night-time. Whenever she has felt upset or nervous, she has preferred to be driven by others. She enjoys driving, and underlines that she is a confident and safe driver. Regarding the risk factors as introduced in the consensus paper from the Swiss memory clinics as mentioned above [19], we only detected the following limitations: The only problem with driving that showed up was that Mrs. L. preferred driving only shorter distances for about one year. Recent medication did not imply any danger for safe driving. There was neither an intake of sedatives nor a newly started administering of ataractics.

The first part of the SAFE, mainly focusing on driving history within the last two years and past medical history as same as recent medication, did not disclose any risk factors for driving.

Mrs. L. and the psychiatrist concluded that there was no obvious hint at reduced driving skills at this stage of diagnostic process. Nevertheless, it was explained that this conclusion could only be preliminary as detailed diagnostic workup was about to come. Final judgement was announced for future consultations after diagnostic workup. The second part of the SAFE that can only be filled in after neuropsychological examination might point toward possibly reduced driving abilities though.

### 2.2. Medical Examination

The neurological examination including rough visual testing was unremarkable. No movement limitation was found in the cervical vertebral column.

#### 2.2.1. Mental State Examination

Mental state examination including questions about orientation, concentration, signs of delusions, fears, obsessive-compulsive thinking or acting, psychomotor disturbances and affective symptoms including suicidality revealed no pathological findings except for her feeling prolonged loneliness after her husband died two years ago. Two months ago, Mrs. L. noticed restlessness and a change in mood, fearing that she may develop dementia. A slight improvement of the restlessness was reported under Citalopram medication. Formal criteria for depression were not fulfilled. There were no signs of day sleepiness. No impulsive behavioral disturbances were present. During consultation, Mrs. L. was irritable and reflected herself on that problem. She reported a thoroughly reduced ability to concentrate once she felt irritated. She described stronger irritability if she was distressed for whatever reason.

#### 2.2.2. Blood Testing

Standard lab work-up was performed by her GP, showing slightly elevated potassium and cholesterol levels without other significant findings.

### 2.3. Summary of the First Appointment and Difficulties that May Arise in Respect to Consultation

#### 2.3.1. Summary

The above history was taken during the first consultation in our Memory Clinic. In summary, we saw an individual with evident short-term memory deficits and slight subjective orientation difficulties (however, objective orientation screening during the consultation showed no abnormalities), that had begun (or were first realized) approximately three months previously; the patient also reported restlessness. There was an unclear history of alcohol consumption that could be potentially relevant. The patient seemed quite irritable during consultation. The patient is an ardent driver, negating difficulties. There were no previous accidents. We did not see any obvious indication that she might pose an immediate threat to herself or others with respect to unsafe driving. We advised the patient to undergo more thorough work-up, including extended neuropsychological testing and brain imaging.

The working diagnosis at this stage of the diagnostic process was “Mild cognitive impairment”.

#### 2.3.2. Pitfalls

We brought up the topic of driving safety at this stage of consultation, but it naturally was subject to preliminary evidence. Although the stated “Mild cognitive impairment” and the history did not include hints at driving problems, but irritability was present. Its significance remained unclear.

Moreover, the unclear history of (potentially relevant) alcohol consumption added to uncertainty. Third-party history was somewhat contradictory, the relationship with the patient’s partner being relatively new. Her daughter’s anxiousness in this respect—the fear that her mother may have driven while drunk, ongoing discussions with her mother on the topic, *etc.*—was taken seriously, albeit the fact that the daughter could not objectively recall the amounts and frequency of consummation. Substantiation remained elusive.

The process of consultation is often not linear, information not always open to clarification. Would it then be indicated to contact the patient and consult her in respect to (new) third-party information to verify on any alcoholic consumption and self-reported short term memory deficits, or, is it more sensible to wait until all tests and examinations have been completed before addressing the subject of driving safety? In the case of Mrs. L., we opted to wait.

### 2.4. Neuropsychological Assessment

After explorative neuropsychological examination during the first appointment, an extensive neuropsychological examination was conducted. Neuropsychological assessment included well-established neuropsychological examination for global cognitive functioning, episodic memory (verbal learning, verbal memory retrieval, nonverbal memory retrieval, and verbal recognition), semantic memory (naming and semantic fluency), attention (processing speed, attention capacity), executive functions (code shifting and cognitive flexibility), and spatial abilities. In addition, depressive symptoms were evaluated using the Beck Depression Inventory (BDI-II) [30,31] and the Geriatric Depression Scale (GDS) [32].

Verbal learning, verbal memory retrieval, and verbal recognition were evaluated with the German version of the Rey auditory verbal learning test (RAVLT) [33], whereas nonverbal memory retrieval was assessed using a subtest (Constructional Praxis II) of the Consortium to Establish A Registry for Alzheimer's Disease (CERAD) [34,35]. Semantic memory was assessed using a 15-item version of the Boston naming test (BNT) [36] and a category fluency task (animal fluency) [37]. Attention was assessed with part A of the trail making test [38,39] and with digit span tasks [40]. Executive functioning was assessed with a number transcoding task from the Dementia Detection Test (DemTect) [41] and with part B of the TMT-B [38,39]. The TMT-B assesses cognitive flexibility, whereas the number transcoding task requires code shifting [42]. Spatial abilities were evaluated using the clock drawing test [43] and a CERAD subtest (constructional praxis I), which requires the duplication of geometric figures. For all of these tests, a higher score reflected better performance, except for the TMT, RAVLT false positives, the number transcoding task and the clock drawing test—here, higher scores reflect lower performance (greater time and number of errors, respectively). The neuropsychological test results are displayed in Table 1.

#### Neuropsychological Test Results

Neuropsychological test results revealed impaired global cognitive abilities (MMSE = 23), poor episodic memory skills, slow processing speed, and impaired cognitive flexibility. In contrast, semantic memory and spatial abilities were unimpaired. Depression screening pointed toward slight depressive symptoms.

The neuropsychologist advised Mrs. L. to refrain from driving until the next consultation with the physician.

### 2.5. Consensus Advice

Regarding possible consequences for Mrs. L.’s driving abilities, the observed cognitive deficits were now deemed to be significant. Especially, Mrs. L.’s very poor performances on the TMT indicated significantly impaired processing speed and cognitive flexibility. Recent studies have shown that a significant association exists between poor TMT-B performances and impaired driving skills [44]. In addition, Mrs. L. showed a reduced MMSE performance, a reduced driving range and reported increased irritability. Consequently, we concluded that the findings might indicate an increased risk for driving.

The second planned consultation with the Memory Clinic physician was now intentionally scheduled earlier (before further work-up could be carried out) because of the results of the neuropsychological examination. We asked both the patient and her daughter to be present. Due to medical confidentiality, we did not inform about any results in advance.

At the time of the appointment, the patient did not show up. Her daughter was present. She was unaware of the whereabouts of her mother. In the waiting period that followed, the patient’s daughter received a phone call. The caller reported that the patient had taken her own car to drive to the Memory Clinic for the appointment—and had driven down a one-way street in the wrong direction, stopping the car abruptly in the process and leaving it unattended. The patient—clearly upset—had asked a bystander to call the daughter.

The situation created a dilemma: on the one hand, there was a now documented risk to the patient and others if she continued driving; on the other hand, confidentiality issues prevented us from immediately communicating the results of the neuropsychological examination to the daughter—indicating dementia as the cause for her driving disability (although the latter would have “delegated” the process of informing the patient to a third party, it would have also allowed the daughter to act to directly hinder further road activity.) In addition, directly informing official parties was not an option, albeit the risk: it was deemed not sufficient enough when weighed against the legal interest of confidentiality.

The missed appointment was rescheduled. With the patient and her daughter then being present, next to the attending physician and the Memory Clinic’s social worker, we advised against further driving. We highlight that in a worst-case-scenario, the driver will not be covered by insurance in the case of an accident, if it is shown that they had been previously informed of the risk incurred by the cognitive disabilities. To help drivers accept our advice to stop driving, we also provided information to seek second opinion on their driving performance by taking part in professional testing at driving schools, but more commonly by official road safety agencies (e.g., the “TÜV” or “DEKRA” organizations in Germany). Unfortunately, when our social worker visited the patient at home three weeks later—to check on the psycho-social situation—Mrs. L. reported that she had continued to drive, although “only for short distances”. Again, it was urgently emphasized that driving, given the cognitive disabilities, was a risk.

The formal working differential-diagnosis at this stage was “mild Alzheimer’s dementia”.

We routinely recommend an MRI. Furthermore, it is common practice to perform CSF-diagnostics in our setting.

### 2.6. Further Work-up

#### 2.6.1. Cerebral MRI

MRI sequences included T1- and T2-weighted imaging, Fluid-attenuated inversion recovery technique (FLAIR) and Suscepibility weighted imaging technique (SWI) —in axial, sagittal and coronal orientations.

The major pathology was patchy bi-hemispheric microangiopathic and lacunar lesions. Some of these lesions showed signal suspicious of microbleeding. There was no sign of more than age-appropriate global or significant mesio-temporal atrophy. We do not routinely perform manual or automated hippocampal volumetry.

#### 2.6.2. Cerebrospinal Fluid

Routine work-up was unremarkable: cell count, glucose and lactate level, albumin and albumin blood/CSF-ratio were normal. No signs of intrathecal synthesis of immunglobulines. (Routine) treponema pallidum screening was negative.

The markers for neurodegenerative disease were, however, pathological.

Total tau protein: 1353 pg/mL (pathological cut-off: ≥450 pg/mL), phosphorylated tau protein: 182 pg/mL (pathological cut-off: ≥61 pg/mL);

β-amyloid 1–42was normal (849 pg/mL cut-off: ≤450 pg/mL), β-amyloid 1–40 was elevated to 35,810 pg/mL. Therefore, the β-amyloid ratio ((β-amyloid 1–42/β-amyloid 1–40) × 10) was pathological (0.24; pathological cut-off: <0.5).

### 2.7. Final Consultation: Summarizing the Diagnostic Results

Results are commonly discussed in a consensus conference at our Memory Clinic. After all, diagnostic information was available, it was concluded that Mrs. L. should be formally diagnosed with mild Alzheimer’s Dementia.

Note that the cerebral microangiopathy may add to the cognitive deficits; we, however, taking the patient’s history into account, did not judge this pathology to be the leading cause of dementia. High blood pressure is a consistent risk factor for cerebral white matter lesions Leukoaraiosis in turn is a risk factor for dementia, thus treatment of hypertension may also be preventive of future additional cognitive decline.

The patient—together with her partner and her daughter—were informed of the diagnosis in a third, concluding, appointment. They were advised in respect to its probable impact on daily living. Moreover, we proposed initiating treatment with a cholinesterase inhibitor. Though the history of alcohol consumption remained patchy, we advised Mrs. L. to reduce any drinking.

Results of neuropsychological examination as cited above, unclear alcohol consumption, emotional irritability and the discovered driving fault were suggestible of reduced driving abilities. The SAFE assessment helped to provide objective results. The patient’s irritability—though more of subjective value—added to our hesitation to recommend driving competence. We discussed possible alternatives of mobility by public transport. The social worker gave detailed information about bus lines and established a possible schedule for Mrs. L. and her daughter for being fetched by her. Our social worker repeated information on diagnosis and driving several times in future appointments, thus contributing to enhanced psychoeducation. She provided aid in dealing with the support and welfare system, which helped Mrs. L. to cope with the situation. Her practical approach alleviated some emotional distress. Additional consultations with the psychiatrist were offered. In our case, Mrs. L. could profit from sensitive contact both with the social worker and with the psychiatrist. The psychiatrist continued outpatient therapy for several more consultations and discussed coping strategies concerning the loss of independent mobility. For example, we could reframe loss of independence by explaining that more contact could be made possible by her daughter fetching her and neighbors accompanying her on the bus trips.

Fortunately, the patient has now consented to our advice and refrained from further driving. She also acknowledged the diagnosis and was willing to be medicated.

## 3. Medical, Ethical and Legal Difficulties

The case reported above touches on several difficulties that frequently occur in the dynamic work-up process of investigating cognitive impairment: history taking may remain incomplete, third-party information may contradict the patient’s impression, and there may be multiple but probable etiological factors. In addition, the tentative impressions from the first consultation may change during the course of more investigations spread over several days or weeks in outpatient settings. Ongoing advice with respect to driving fitness must therefore explain the potential negative impact of cognitive deficits on driving competence, and be delivered in a sensitive, professional manner that is neither too strict nor too uncritical. Consultation on driving competence for people with cognitive deficits is therefore challenging, due to medical, ethical and legal difficulties.

In the following, we reflect on these with respect to the case presented here.

### 3.1. Medical Difficulties

The completeness of this patient’s history at different time-points of the work-up process challenged an early substantiated differential-diagnosis.

Although the patient reported subjective cognitive complaints, information available at the first consultation did not warrant advising against driving—even given information available later as to the crucial question about alcohol consumption. It remains unclear to what extent (non-pathological) alcohol consumption even within legal limits may increase the risk for road drivers with initial cognitive deficits—although it is deemed to be relevant.

After extensive neuropsychological examination, it was clear that there were significant cognitive deficits, not only in the mnestic domains but also slow processing speed and impaired cognitive flexibility. Latter results especially prompted us to advise the patient to refrain from further driving until the more complete work-up would provide a definite diagnosis. Note, however, that there is to date no scientifically validated exact correlation between neuropsychological impairment, especially given milder deficits, and the degree of on-the-road driving safety as illustrated above (see Section 1.1). Although desirable, memory clinics do not have the resources to provide “real-life” modeling of driving abilities to aid the consultation process.

Further points are worth highlighting. The effects of short-time memory dysfunction alone on driving abilities are not clear and further studies are warranted. In fact, Ernst *et al.* [6] point out that for a remarkable proportion of drivers with Alzheimer’s disease, driving abilities remain unchanged, even in mild to moderate stages of dementia. In contrast, low performance on the TMT-B test has been shown to be a good marker for driving problems [31].

Our intent to advise against further driving was substantiated by the fact that the patient exhibited at-risk driving behavior by driving in the wrong direction along a one-way-street just prior to the appointment. However, even this episode can be questioned as to be entirely attributed to cognitive deficits as such. An alternative explanation for this singular event (her driving history up to this time-point was reported to have been unremarkable) may have been that Mrs. L. was experiencing stress just before the visit in our Memory Clinic. She knew that we would be discussing results relevant to her driving abilities. We, however, opted to interpret it as showing that additional stress leads to decompensation given underlying cognitive disabilities. In addition, Mrs. L. showed a reduced MMSE performance, a reduced driving range and reported increased irritability. Consequently, we concluded that the findings might indicate an increased risk for driving.

It is important to note that the patient’s history and reports from relatives or other informants play a pivotal role in the diagnostic process—their credibility and ability to report on symptoms with sufficient precision influences potentially embarrassing information (e.g., alcohol consumption). The presence or the lack of information from these sources heavily influences our decision-making. Technical work-up is not always possible. In this patient’s case, it substantiated our diagnosis.

### 3.2. Ethical Difficulties

At first, the more philosophical question arises if one can adequately translate results from neuropsychological examinations or other technical procedures to the intricate functioning of an individual. Additionally, there remain ethical liabilities in the consultation process.

We have to make (subjective) decisions when and when not to discuss the potential consequences of their cognitive disabilities with our patients. Issues such as the ability to participate in social activities, given driving restrictions, must be taken into account.

Apart from that, we have an obligation to society (and to the patient) to protect it (him or her) from unwarranted risk. Negating confidentiality issues here, sometimes it will not be possible to do so without involving the patient’s relatives or other third-parties. This, however, can be ethically difficult: Are we allowed to involve them as chaperones, responsible for the road safety (e.g., hiding the car keys)? In the authors’ experience, relatives are often left alone with these problems—outside the formal consultation process of a memory clinic.

Moreover, there are some formal aspects such as those related to insurance issues. We may be “forced” to inform patients to protect ourselves from recourse in the case of an accident. On the other hand, delegating responsibility to “more” official institutions is seldom an option. However, for example, these may offer more reliable practical driving safety testing; legislation in most countries is such that the substantial costs for these procedures have to be covered by the testee.

Unfortunately, few studies explicitly examine these issues and consequently inform us how to best advise elderly patients with cognitive deficits that still drive.

### 3.3. Legal Difficulties

The question arises whether relatives or other third-parties (including legal guardians) have a legal responsibility to guarantee that cognitively impaired individuals that have been deemed unfit to drive do not drive. Legislation is unclear here and court rulings (in the German context) do not give a clear picture. Loved ones are also those that are most often involved and worried about dangers and risk. They are also those that may find it difficult to discuss matters such as the necessity to stop driving with the patient.

Relatives may, however, remain unaware of an underlying problem given that they are not informed by the patient. Medical confidentiality hinders any direct communication between the physician and third-parties without the patients consent. Medical confidentiality is a predominantly important legal interest. In the German system, violating confidentiality is limited to situations in which there is imminent and severe risk for the patient (or others) that cannot be avoided.

We did not deem our patient’s situation to not take the above hurdle, even given her driving down the one-way street in the wrong direction. We acknowledge that other physicians may have decided otherwise. A (legal) compromise may be to consult relatives that one does not see a patient to be fit driving, without going into medical detail as to why.

## 4. Conclusions

There will be many more constellations, necessitating, at times, an even more complicated balancing of medical, ethical and legal questions when managing a patient with cognitive deficits, in terms of driving fitness at the Memory Clinic.

There are still a lot of questions unanswered. Doctors face questions about an adequate and practical work-up in the process of diagnosis and determining driving fitness. On the other hand, they have to weigh up the potential consequences or impact of their decision making in the consultation process. It also remains unclear how society should and must deal with restrictions on driving with unfit older drivers in an ageing society, balancing personal freedom of community mobility with individual and public safety. These topics must be addressed in the near future.

The German context with limited access to on-road-driving assessments poses a special ethical dilemma that seems worthwhile discussing. Future studies should focus on the evaluation of assessment instruments like the SAFE. Future research endeavors should generally include on-road-testing for people with cognitive deficits as the gold standard as well as comparing outcomes with a healthy control group. From our case report, studies should additionally evaluate the consensus process in our multiprofessional team. The authors are convinced that the time has come for the implementation of a standardized assessment procedure that is well evaluated and yet can be individualized to the needs of patients showing early cognitive decline. Health professionals need to be reflective in their clinical reasoning and approaches when managing drivers with cognitive deficits—asking questions like “Whom do we want to come across or encounter when we are driving ourselves ?” or “How do we want to be advised on our driving abilities if we ever suffer from cognitive deficits?”. In the end, we argue for a respectful and ethically well-balanced consultation service at the Memory Clinics.

## Figures and Tables

**Table 1:** Neuropsychological test results.

Neuropsychological Domain		Test	Raw Score	z-Score
Global cognitive status		MMSE	23	impaired
Episodic memory	verbal learning	RAVLT trial 1	3	−1.3
	verbal learning	RAVLT trial 2	3	−2.1
	verbal learning	RAVLT trial 3	5	−1.5
	verbal learning	RAVLT trial 4	4	−2.4
	verbal learning	RAVLT trial 5	5	−2.3
	verbal learning	RAVLT Σ trials 1–5	20	−2.3
	verbal retrieval	RAVLT free recall	0	−2.4
	nonverbal retrieval	Constructional Praxis II	0	−2.8
	recognition	RAVLT recognition	10	−1.0
	recognition	RAVLT false positives	15	−8.4
Semantic memory	naming	BNT	15	0.9
	semantic fluency	animal fluency	16	−0.7
Attention	processing speed	TMT-A (seconds)	87	−2.1
	word span	VLMT–D1	3	−1.3
	digit span	repeating numbers forward	6	0,1
Executive function	code shifting	number transcoding (errors)	1	not impaired
	cognitive flexibility	TMT-B (seconds)	241	−2.4
Spatial abilities	free drawing	Clock drawing test (errors)	0	not impaired
	copy	Constructional Praxis I	11	0.8
Depressiveness		GDS	3	normal mood
		BDI-II	16	slightly depressive

MMSE = Mini Mental State Examination; RAVLT = auditory verbal learning test; BNT = Boston naming test; TMT-A = Part A of the trail making test; TMT-B = Part B of the trail making test; GDS = Geriatric Depression; BDI = Beck Depression Inventory.
